# Quality of life among the Arab population two years after COVID-19 pandemic

**DOI:** 10.1186/s12889-023-16171-z

**Published:** 2023-06-30

**Authors:** Mohamed Mostafa Tahoun, Horeya M. Ismail, Osman Abubakar Fiidow, Rasha Ashmawy, Esraa Abdellatif Hammouda, Iffat Elbarazi, Ramy Mohamed Ghazy

**Affiliations:** 1 Department of Epidemiology, High Institute of Public Health, Alexandria University, Alexandria, Egypt; 2 High Institute of Public Health, Alexandria University, Alexandria, Egypt; 3 School of Public Health and Research, Mogadishu, Somali National University, Mogadishu, Somalia; 4 Department of Clinical Research, Maamora Chest Hospital, MoHP, Alexandria, Egypt; 5 Clinical Research Department, El-Raml Pediatric Hospital, MoHP, Alexandria, Egypt; 6 Institute of Public Health, College of Medicine &Health Sciences, United Arab Emirates University, Al Ain, UAE; 7 Tropical Health Department, High Institute of Public Health, Alexandria University, Alexandria, Egypt

**Keywords:** COVID-19, Quality of life, Arab Countries, Quality of life domains, Country income level, Chronic diseases, Impact of COVID-19

## Abstract

**Background:**

The coronavirus disease 2019 (COVID-19) pandemic has resulted in severe consequences worldwide. Our study aims to assess the quality of life (QoL) domains and its determinants among the general population in Arab countries after two years of the COVID-19 pandemic. Methods: An anonymous online cross-sectional survey using the short version of World Health Organization QoL (WHOQOL-BREF) instrument was distributed among Arab adults in 15 Arab Countries.

**Results:**

A total of 2008 individuals completed the survey. Amongst them, 63.2% were 18–40 years and 63.2% were females, 26.4% had chronic disease, 39.7% confirmed having contracted COVID-19, and 31.5% had experienced the unfortunate loss of relatives due to COVID-19. The survey revealed that 42.7% reported good physical QoL, 28.6% were satisfied with psychological QoL, 32.9% had a sense of well-being in the social domain, and 14.3% had good QoL in the environmental domain. The predictors of physical domains were as follows: being a male (β = 4.23 [95%CI 2.71, 5.82]), being from low-middle income country (β = -3.79 [95%CI -5.92, -1.73]) or being from high-middle-income country (β = -2.95 [95%CI -4.93, -0.92]), having a a chronic disease (β = -9.02 [95%CI -10.62,-7.44]) having a primary/secondary education (β = -2.38 [95%CI -4.41, -0.054]), number of years of work experience ≥ 15 years (β = 3.25 [95%CI 0.83, 5.73]), income-per-capita [ranged from (β = 4.16 [95%CI -5.91, -2.40]) to (β = -11.10 [95CI%, -14.22, -8.11])], a previous COVID-19 infection (β = -2.98 [95%CI -4.41, -1.60]), and having relative died from COVID-19 (β = -1.56 [95%CI -3.01, -0.12]). The predictors of psychological domain were having a chronic disease (β = -3.15 [95%CI -4.52, -1.82]), a postgraduate education (β = 2.57 [95% CI 0.41, 4.82]), number of years of work experience ≥ 15 years (β = 3.19 [95%CI 1.14, 5.33]), income-per-capita [ranged from (β = -3.52 [95%CI -4.91, -1.92]) to (β = -10.31 [95%CI -13.22, -7.44])], and a previous COVID-19 infection (β = -1.65 [95%CI -2.83, -0.41]). The predictors of social domain were being a male (β = 2.78 [95%CI 0.93, 4.73]),  being single, (β =-26.21 [-28.21, -24.32]), being from a low-income country (β = 5.85 [95%CI 2.62, 9.13]), or from a high**-**middle-income country (β = -3.57 [95%CI -6.10, -2.12]), having a chronic disease (β = -4.11 [95%CI -6.13, -1.11]), and income-per-capita [ranged from (β = -3.62 [95%CI -5.80, -1.41]) to (β = -11.17 [95%CI -15.41, -6.92])]. The predictors of environmental domain were being from a low-middle-income country (β = -4.14 [95%CI -6.90, -1.31), from a high-middle-income country (β = -12.46 [95%CI -14.61, -10.30]), or from a low-income-country (β = -4.14 [95%CI, -6.90, -1.32]), having a chronic disease (β = -3.66 [95%CI -5.30, -1.91]), having a primary/secondary education (β = -3.43 [95%CI -5.71, -1.13]), being not working (β = -2.88 [95%CI -5.61, -0.22]), income-per-capita [ranged from (β = -9.11 [95%CI -11.03, -7.21] to (β = -27.39 [95%CI -31.00, -23.84])], a previous COVID-19 infection (β = -1.67 [95%CI -3.22, -0.21]), and having a relative who died from COVID-19 (β = -1.60 [95%CI -3.12, -0.06].

**Conclusion:**

The study highlights the need for public health interventions to support the general population in the Arab countries and mitigate its impact on their QoL.

**Supplementary Information:**

The online version contains supplementary material available at 10.1186/s12889-023-16171-z.

## Introduction

The outbreak of the novel coronavirus disease 2019 (COVID-19) in China in late December 2019 rapidly evolved into a public health emergency of International Concern (PHEIC). With the massive global-wide spread of the disease, the World Health Organization (WHO) announced the outbreak of COVID-19 as a global pandemic on the 12^th^ of March 2020 [1]. The COVID-19 pandemic has had different patterns of morbidity and mortality across countries [2]. Until May 24, 2023, the number of confirmed of severe acute respirtaory syndrom coronavirus 2 (SARS-CoV-2) cases globally reached approximately 766.90 million, leading to approximately 6.93 million fatalities. In the Eastern Mediterranean Region (EMR), there were approximately 23.4 million confirmed cases and approximately 351.23 deaths. The number of confirmed COVID-19 cases varied widely across Arab countries with Iraq recorded the highest number of confirmed cases, reaching approximately 2.3 million, while Tunisia recorded the highest number of COVID-19 related deaths among Arab countries [3].

Like other countries, authorities in the Arab countries, implemented unprecedented measures such as community lockdowns, curfews, physical distancing, travel restrictions, quarantines, and cancellation of social events [4, 5]. These measures disrupted social norms and caused distress for many people, with a devastating impact on the economy [6]. Furthermore, the measures taken to mitigate the spread of COVID-19 have had detrimental effects on the mental health of populations in many countries. The pandemic and the associated restrictions have resulted in an increase in the prevalence of mental health issues, such as depression, anxiety, and various other psychological problems [7, 8]. Existing evidence suggests that various sociodemographic factors played a role in determining the vulnerability of populations to psychological distress during the COVID-19 pandemic. Factors such as gender, age, educational level, social support, experience with COVID-19 infection, length of isolation, amount of exposure to media, and personal resources including personality traits, and income level have been identified as potential contributors [9, 10]. This negative impact on mental health, in turn, significantly affected the quality of life (QoL) of the general population [11, 12].

The terms” QoL”, "health-related quality of life" (HRQoL), and "health" are occasionally used interchangeably [13]. As per the WHO, health is defined as a state of complete well-being that includes physical, mental, and social dimensions. It goes beyond the mere absence of disease or infirmity [14]. According to the definition provided by Bowling et al. [15], “HRQoL” encompasses the optimal levels of mental, physical, and social functioning. This includes various aspects such as one's ability to perform different roles (e.g., work, parenting, career), maintain relationships, and perceive own health, fitness, life satisfaction, and overall well-being. Subsequently, the term “QoL” was introduced to shift the focus from solely relying on the clinician's assessment to incorporating the patients' own expressions of their preferences and values [16]. So that, it is important to recognize that QoL and HRQoL are not synonymous terms. While both concepts relate to individuals' well-being, there are distinctions between them [17]. QoL is a multifaceted and a comprehensive concept that encompasses various dimensions influenced by individual perceptions. These include the physical, psychological, social, and environmental domains. The WHO defines QoL as an individual's subjective evaluation of their position in life, taking into account the cultural and value systems within which they exist [16]. The physical health domain encompasses various aspects related to an individual's well-being. It provides insight into a person's physical functioning and overall health status, assessing their ability to move freely, carry out everyday tasks, maintain energy levels, manage pain, and experience restful sleep. The psychological domain comprises various aspects that contribute to an individual's psychological well-being. It involves measuring factors such as self-image, negative thoughts, positive attitudes, self-esteem, mentality, learning ability, memory concentration, religion, and mental status [18]. As for, the social relationships domain of QoL, it encompasses aspects of an individual's social interactions and connections. It includes inquiries about personal relationships, social support, and sex life. Finally, the environmental domain QoL encompasses various aspects related to an individual's physical surroundings and the resources available to them. It includes factors such as financial resources, safety, access to healthcare and social services, living conditions, opportunities for learning and skill development, recreational options, the general environment (including noise and air pollution), and transportation [19]. Indeed, the measures and restrictions implemented to mitigate the transmission of COVID-19 have had a substantial impact on the general well-being and various dimensions of QoL among the population. The pandemic has brought about significant changes to daily life, social interactions, work, education, and leisure activities [20].

The negative impact of the COVID-19 pandemic has varied across Arab countries, influenced by diverse socio-cultural, environmental, economic, and political contexts. In regions with conflict zones and humanitarian crises, studies have highlighted existing challenges faced by populations already burdened with cumulative stressors and traumas. Among these populations the effects of COVID-19 imposed worsened health conditions, exacerbated psychological effects on the population, and increased their vulnerability [21]. In contrast, high-income Arab countries have reported better coping mechanisms during the pandemic among their populations, resulting in mild to moderate levels of anxiety and stress with minimal effect on QoL [22, 23]. The objective of this study was to assess the domains of QoL and to understand the factors that influence them among the general population in Arab countries after two years of the COVID-19 pandemic. Specifically, the study aimed to investigate the determinants of each QoL domain, including the socio-cultural, economic, and specific COVID-19 related factors.

## Methods

### Study design and participants

This study employed across-sectional design using an online survey to collect data from individuals aged 18 years or older residing in Arab countries. The target population consisted of individuals who were using various social media platforms and had access to the internet through smartphones or computers.

### Sample size calculation

Using G power, 3.19.1.4, based on a previous study that found that the mean total QoL score among Egyptian was 2.3 ± 0.6 [24], alpha error of 0.05, power of 80%, and size effect of 0.83 (based on the pilot study the mean total QoL score 2.8), the minimum required sample size was 14/country.

### Data collection

The survey for this study was distributed online through different social media platforms like Facebook, Twitter, WhatsApp, and Telegram. The data collection period took place between February 22 and March 26, 2022. The survey gathered information on a range of factors, including sociodemographic characteristics such as age, gender, and education, crowding index (The crowding index is determined by dividing the number of individuals living in a particular dwelling by the number of rooms available in that dwelling), medical history and presence of chronic diseases, previous COVID-19 infection, COVID-19 vaccination status, and experiences with COVID-19-related deaths among relatives. The COVID-19 vaccination status can be classified into three categories: fully vaccinated, partially vaccinated, and not vaccinated. Fully vaccinated refers to individuals who have completed the primary series of vaccination. Partially vaccinated refers to individuals who have received the first dose of the vaccine and are awaiting the second dose. Not vaccinated refers to individuals who have not received any doses of the COVID-19 vaccine.

In order to evaluate the QoL in this study, we utilized the validated short version of the World Health Organization Quality of Life (WHOQOL-BREF) instrument in Arabic [25], English [19], and French [26]. WHOQOL-BREF consists of four domains that encompass different aspects of QoL: physical, psychological, social, and environmental. The instrument consists of 26 items, of which two items evaluate general QoL and general health, and 24 items assess QoL in the four domains mentioned earlier, physical (7 items), psychological (6 items), social relationship (3 items), and environmental domain (8 items) [27, 28]. The assessment of QoL using the “WHOQOL-BREF” tool involves participants providing responses to each question on a 5-point Likert scale. The scale ranges from 1 to 5, where 1 represents options such as "very poor," "very dissatisfied," "none," or "never," and 5 represents options such as "very good," "very satisfied," "extremely," or "always." After collecting responses to the questionnaire, the scores for each of the four domains were calculated by summing the scores of the respective items within each domain. These domain scores were then transformed to a positive 0–100 scale, with higher scores indicating a better QoL. The mean QoL domain scores for the general population were estimated to be as follows: physical health 73.5 ± 18.1, psychological 70.6 ± 14.0, social relationships 71.5 ± 18.2, and environmental quality of life 75.1 ± 13.0. Participants who scored above these values were classified as having good QoL, while those who scored below these values were considered to have poor QoL [28]. The countries were categorized based on their income per capita into low-income, low-middle-income, high-middle-income, and high-income, according to the World Bank Classification.

### Study outcome assessment

The primary objective of this study was to evaluate the domains of QoL and to examine the factors that impact them among the general population of Arab countries following a two-year period since the onset of the COVID-19 pandemic.

#### Statistical analysis

Categorical variables were summarized using frequency and proportion, while continuous variables were assessed for normality through visual inspection and the Kolmogorov–Smirnov test. Normally distributed continuous variables were described using the mean and standard deviation (mean ± SD). Inferential statistics were conducted using independent t-tests and ANOVA, to compare groups and identify any significant differences. Regression modeling was also employed to investigate the factors influencing different domains of QoL. Prior to regression analysis, multicollinearity was checked using tolerance and variance inflation factor to ensure the independence of predictor variables. All statistical analyses were performed using two-tailed tests, with a significance level set at 0.05. The IBM SPSS software (Statistical Packages for Social Sciences) version 27 for Windows and STATA 14.2 were used to conduct the data analysis.

## Results

**Fig. 1:**
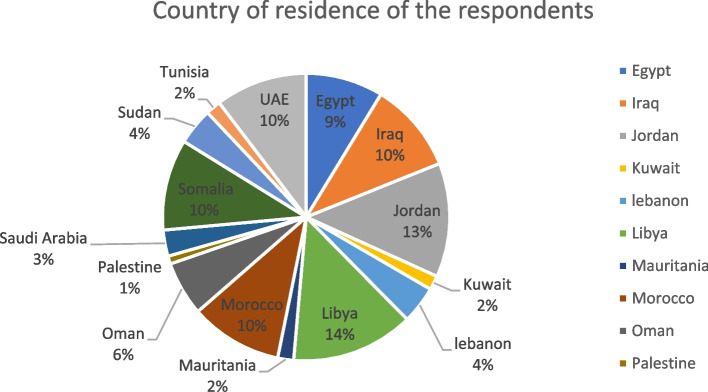
Country of residence of the respondents

The survey included a total of 2008 participants from 15 Arab countries. The distribution of participants from each country is visually represented in Fig. 1. The questionnaire was circulated in three languages, with the majority of participants answering in Arabic (77.3%), followed by English (15.4%) and French (7.3%). Nearly three-fifths of respondents were mostly young aged 18–40 years (63.2%), 63.2% were females, 57.2% were married, 88.6% resided in urban areas, 36.6% lived in high-middle-income countries, 52.0% had sufficient income, and 89.4% had a crowding index of more than 1. Furthermore, more than half of the participants had a university degree (53.1%), 39.4% were not working, and 42.3% had less than five years of work experience. In addition, 26.4% had chronic diseases, 39.7% had confirmed COVID-19 infection, 48.3% were fully vaccinated, and 31.5% had relatives who died due to COVID-19 (Table 1).

**Table 1:** Socio demographic characteristics of the study participants (*n* =2008)

Variables		Frequency *N* = 2008	%	95% CI
**Language**	Arabic	1553	77.3%	[75.5; 79.2]
	English	310	15.4%	[0.39; 17.1]
	French	145	7.3%	[6.1; 8.4]
**Sex**	Female	1269	63.2%	[61.1; 65.3]
	Male	739	36.8%	[34.7; 38.9]
**Age**	18- 40 years	1269	63.2%	[61.1; 65.3]
	≥ 40	739	36.8%	[34.7; 38.9]
**Marital status**	Married	1149	57.2%	[55.0; 59.4]
	Single	859	42.8%	[40.6; 44.9]
**Residence**	Rural / remote area	229	11.4%	[10.1; 12.9]
	Urban	1779	88.6%	[87.1; 89.9]
**Country income level**	Low	294	14.7%	[13.1; 16.3]
	Low-middle income	539	26.8%	[24.9; 28.8]
	High-middle income	735	36.6%	[34.5; 38.8]
	High income	440	21.9%	[20.1; 23.8]
**Income per capita**	Enough	1045	52.0%	[49.8; 54.3]
	Enough and save	387	19.3%	[17.6; 21.1]
	Not enough and borrowing large sums	96	4.8%	[3.9; 5.8]
	Not enough and borrowing small amounts	352	17.5%	[15.9; 19.3]
	Not enough, and he/she is in debt, and he/she cannot fulfil the debt	128	6.4%	[5.4; 7.5]
**Crowding index**	Less than 2	1535	76.4%	[74.5; 78.3]
	From 2–3	439	21.9%	[20.1; 23.7]
	Above 4	34	1.7%	[1.2; 2.4]
**Education**	Illiterate	15	0.7%	[0.4; 1.2]
	Reads and writes	36	1.8%	[1.3; 2.5]
	Primary	30	1.5%	[1.0; 2.1]
	Preparatory	70	3.5%	[2.7; 4.4]
	Secondary	296	14.7%	[13.2; 16.4]
	University graduate	1067	53.1%	[50.9; 55.3]
	Postgraduate	494	24.6%	[22.7; 26.7]
**Working sector**	Governmental sector	626	31.2%	[29.2; 33.3]
	Private sector	590	29.4%	[27.4; 31.4]
	Not working	792	39.4%	[37.3; 41.6]
**Years of work experience**	Less than 5 years	850	42.3%	[40.2; 44.5]
	5- 9 years	301	15.0%	[13.5; 16.6]
	10—14 years	259	12.9%	[11.5; 14.4]
	≥ 15 years	598	29.8%	[27.8; 31.8]
**Having chronic diseases**		530	26.4%	[24.5; 28.4]
**Confirmed infection with COVID-19**		798	39.7%	[37.6; 41.9]
**COVID-19 vaccination**	Fully vaccinated	969	48.3%	[46.1; 50.5]
	Not vaccinated	549	27.3%	[25.4; 29.4]
	Partially vaccinated	187	9.3%	[8.1; 10.1]
	Received the booster dose	303	15.1%	[13.6; 16.7]
**Had relative died due to COVID-19**		632	31.5%	[29.5; 33.6]

Table 2 displays the means and standard deviations for the total and domain scores of the “WHOQOL-BREF: questionnaire**.** The overall average score for QoL was 63.1 ± 13.6. The mean scores for general health and general QoL were 3.6 ± 0.9 and 3.7 ± 1.0, respectively. Among the participants, 42.7% reported good QoL in the physical domain, 28.6% in the psychological domain, 32.9% in the social domain, and 14.3% in the environmental domain.

**Table 2:** Summary of the quality-of-life scores from WHOQoL-BREF domains

Item	Min	Max	Mean ± SD	Cut-off point	Good n (%)
Physical	3.6	100.0	68.3 ± 16.9	73.5 ± 18.1	857(42.7)
Psychological	12.5	95.8	60.9 ± 13.7	70.6 ± 14.0	574(28.6)
Social relation	0.0	100.0	56.5 ± 24.1	71.5 ± 18.2	660(32.9)
Environment	0.0	100.0	57.1 ± 19.1	75.1 ± 13.0	287(14.3)
General health	1	5	3.6 ± 0.9	-	
General QoL	1	5	3.7 ± 1.0	-	
Total score	27.0	113.0	63.1 ± 13.6		

The bivariable analysis between sociodemographic factors and QoL among the general population revealed statistically significant mean differences in all four QoL domains for several sociodemographic variables. Significant differences were found in the mean scores of psychological, social, and environmental health domains based on age. Participants aged 40 years and above had higher mean scores compared to those below 40 years of age. Significantly higher mean scores in the physical, social relations, and environmental domains were observed among male participants compared to females. Individuals residing in urban areas exhibited significantly higher mean scores in the environmental domain compared to those living in rural areas (55.9 ± 19.1 vs. 59.0 ± 18.9,* p* = 0.001). Significant mean differences were observed in the psychological, social relations, and environment domains based on marital status. Married participants demonstrated higher scores in terms of their psychological, social, and environmental health compared to unmarried individuals. Significant mean differences were found across different educational levels in all domains of QoL. Participants with a postgraduate degree had higher scores in all QoL domains compared to individuals with other levels of education. There were significant mean differences in all QoL domains across different working sectors. Participants working in the governmental sector had higher mean scores compared to individuals in other sectors. Additionally, there were significant mean differences in all four QoL domains based on the variable of profession. Being employee/worker had a higher mean score compared to those not working or housewives. There were significant mean differences in all QoL domains, except for social relations, based on comorbidities. Participants without comorbidities had higher mean scores compared to those who had comorbidities. There were significant mean differences in all QoL domains, except for the physical domain, based on years of work experience. Participants with less than 5 years of work experience had higher mean scores compared to those with having more years of experience (Table 3).

**Table 3:** Bivariable analysis of sociodemographic factors and quality of life

Variable	Physical domain	Psychological domain	Social relationship	Environment
	Mean ± SD	Mean ± SD	Mean ± SD	Mean ± SD
**Age**				
< 40 years	68.8 ± 16.6	60.3 ± 13.9	52.4 ± 24.4	55.9 ± 19.1
≥ 40 years	67.6 ± 17.3	61.9 ± 13.2	63.5 ± 21.7	59.0 ± 18.9
*P*^*a*^	0.141	0.012	0.001	0.001
**Gender**				
Female	66.1 ± 17.0	60.4 ± 13.7	53.5 ± 24.1	55.9 ± 19.3
Male	72.1 ± 15.9	61.7 ± 13.5	61.5 ± 23.2	59.2 ± 18.5
*P*^*a*^	0.001	0.042	0.001	0.001
**Geographical area**				
Urban	68.6 ± 16.6	61.0 ± 13.5	56.8 ± 24.1	57.8 ± 18.7
Rural	66.4 ± 18.7	59.9 ± 14.9	54.0 ± 23.4	51.5 ± 20.8
*P*^*a*^	0.065	0.264	0.092	0.001
**Marital Status**				
Married	68.5 ± 16.5	61.8 ± 13.2	68.4 ± 19.8	58.8 ± 18.6
Single	68.1 ± 17.3	59.8 ± 17.1	40.5 ± 19.5	54.7 ± 19.5
*P*^*a*^	0.660	0.001	0.001	0.001
**Education**				
Less than primary	68.8 ± 16.9	59.4 ± 13.7	51.4 ± 24.7	55.7 ± 19.9
Primary/Secondary	66.2 ± 17.9	58.2 ± 14.0	54.8 ± 23.0	53.4 ± 19.1
University level	68.9 ± 16.3	61.9 ± 13.6	58.0 ± 24.0	58.4 ± 18.4
Postgraduate Level	69.3 ± 16.7	63.1 ± 11.9	60.6 ± 23.8	60.4 ± 19.1
*P*^*b*^	0.028	0.001	0.001	0.001
**Working sector**				
Government sector	70.1 ± 15.9	62.9 ± 12.9	63.2 ± 21.8	59.1 ± 18.0
Private sector	70.4 ± 16.3	61.1 ± 13.9	59.3 ± 23.6	58.5 ± 19.1
Not working	65.4 ± 17.6	59.2 ± 13.9	49.1 ± 24.1	54.4 ± 24.1
*P*^*b*^	0.001	0.001	0.001	0.001
**Profession**				
Employee/worker	70.2 ± 15.1	61.7 ± 13.5	59.9 ± 23.2	58.5 ± 18.5
Not working/housewife	64.7 ± 17.9	59.3 ± 13.8	50.1 ± 24.4	54.4 ± 19.8
*P*^*a*^	0.001	0.001	0.001	0.001
**Comorbidities**				
No	70.9 ± 15.5	61.7 ± 13.2	56.9 ± 23.8	58.0 ± 18.8
Yes	61.2 ± 18.4	58.6 ± 14.6	55.1 ± 26.6	54.4 ± 19.7
*P*^*a*^	0.001	0.001	0.145	0.001
**Years of work experience**				
≥ 15 years	67.4 ± 16.9	59.7 ± 14.1	47.7 ± 23.6	55.0 ± 19.3
5–10 years	69.0 ± 16.4	60.1 ± 14.5	59.1 ± 24.1	55.4 ± 20.1
10–15 years	67.5 ± 16.6	60.1 ± 12.2	63.1 ± 21.7	58.1 ± 18.1
Less than 5 years	69.6 ± 17.1	63.4 ± 12.9	64.8 ± 21.3	60.4 ± 18.2
*P*^*b*^	0.064	0.001	0.001	0.001

*P*^*a*^ = T test *P*-value

*P*^*b*^ = ANOVA *P*-value

### Comparisons between COVID19 infection factors and quality of life

Table 4 demonstrates significant mean differences in various QoL domains based on different factors. Regarding previous diagnoses of COVID-19 infection, significant mean differences were observed in the physical, psychological, and environmental domains. Participants without a previous COVID-19 diagnosis had higher mean scores in the physical (69.8 ± 16.4 vs 66.1 ± 17.2, *p* = 0.001), psychological (61.5 ± 16.0 vs 60.0 ± 16.5, *p* = 0.021), and environmental (58.0 ± 19.0 vs 55.6 ± 19.2, *p* = 0.006) domains compared to those who had been diagnosed with COVID-19. In terms of COVID-19 vaccination, significant mean differences were found in the social and environmental domains. Participants who were not fully vaccinated had higher mean scores in social (55.6 ± 24.1 vs 59.2 ± 23.6, *p* = 0.004) and environmental (55.8 ± 19.0 vs 61.1 ± 18.8, *p* = 0.001) domains compared to those who were fully vaccinated. Furthermore, participants who had relatives that died due to COVID-19 exhibited significantly lower mean scores. Specifically, the score of physical domain (69.2 ± 16.6 vs 66.4 ± 17.3, *p* = 0.001), the social domain (57.4 ± 23.9 vs 54.4 ± 24.3, *p* = 0.011), and environmental domian (58.0 ± 19.3 vs 55.0 ± 18.3, *p* = 0.001).

**Table 4:** Bivariate analysis of COVID19 factors and quality of life

Variable	Physical domain	Psychological domain	Social relationship	Environmental
	Mean ± SD	Mean ± SD	Mean ± SD	Mean ± SD
Pervious COVID 19 infections				
No	69.8 ± 16.4	61.5 ± 16.0	56.9 ± 24.0	58.0 ± 19.0
Yes	66.1 ± 17.2	60.0 ± 16.5	55.8 ± 24.1	55.6 ± 19.2
*p*	0.001	0.021	0.381	0.006
COVID 19 Vaccination				
Full vaccinated	67.9 ± 16.9	60.7 ± 13.9	55.6 ± 24.1	55.8 ± 19.0
Not Vaccinated	69.6 ± 16.8	61.5 ± 12.8	59.2 ± 23.6	61.1 ± 18.8
*p*	0.051	0.276	0.004	0.001
A relative died due to COVID 19				
No	69.2 ± 16.6	61.1 ± 13.8	57.4 ± 23.9	58.0 ± 19.3
Yes	66.4 ± 17.3	60.5 ± 13.2	54.4 ± 24.3	55.1 ± 18.3
*p*	0.001	0.341	0.010	0.001

*Independent T-test, SD* Standard deviation

### Predictors of different domains of QOL

The multilinear regression analysis demonstrated that several socio-demographic and economic factors significantly influenced QoL domains. Factors associated with physical domains were male gender (β = 4.23 [95%CI 2.71, 5.82], low-middle income country (β = -3.79 [95%CI -5.92, -1.73]), living in a high middle income country (β = -.95 [95%CI -4.93, -0.92]), having a chronic disease (β = -9.02 [95%CI -10.62, -7.44]), primary/secondary education (β = -2.38 [95%CI -4.41, -0.05]), years of work experience ≥ 15 years (β = 3.25 [95%CI 0.83, 5.73]), income per capita [ranged from (β = -4.16 [95%CI -5.91, -2.40]) to (β = -11.10 [95CI%, -14.22, -8.11)], a previous COVID-19 infection (β = -2.98 [95%CI -4.41, -1.61]), and having relative who died from COVID-19 (β = -1.56 [95%CI -3.01, -0.12]). Predictors of psychological domain were having a chronic disease (β = -3.15 [95%CI -4.52, -1.82]), postgraduate education (β = 2.57 [95%CI 0.41, 4.82]), number of years of work experience ≥ 15 years (β = 3.19 [95%CI 1.14, 5.33]), income-per-capita [ranged from (β = -3.52 [95%CI -4.91, -1.92]) to (β = -10.31 [95%CI -13.22, -7.44])], and a previous COVID-19 infection (β = -1.65 [-2.83, -0.41]).

Predictors of social domain were being male (β = 2.78 [95%CI 0.93, 4.73]), being single, (β = -26.21 [-28.21, -24.32]), living in a low-income country (β = 5.85 [95%CI 2.62, 9.13]), living in a high**-**middle-income country (β = -3.57 [95%CI -6.10, -2.12]), having a chronic disease (β = -4.11 [95%CI -6.13, -1.11]), and income-per-capita ranged from (β = -3.60 [95%CI -5.80, -1.41]) to (β = -11.17 [95%CI -15.41, -6.92]).

Predictors of environmental domain were living in a low-middle-income country (β = -4.14 [95%CI -6.90, -1.31]), living in a high-middle-country (β = -12.46 [95%CI -14.61, -10.30]), living in a low-income country (β = -4.14 [95%CI, -6.90, -1.32]), having a chronic diseases (β = -3.66 [95% -5.30, -1.91]), primary/secondary education (β = -3.43 [95%CI -5.71, -1.13]), being not working (β = -2.88 [95%CI -5.61, -0.22]), income-per-capita [ranged from (β = -9.11 [95%CI -11.03, -7.21] to (β = -24.63 [95%CI -27.39, -31.00])], having a previous COVID-19 infection (β = -1.67 [95%CI -3.22, -0.21]), and having a relative who died from COVID-19 (β = -1.60 [95%CI -3.12, -0.06] (Table 5).

**Table 5:** Predictors of different domains of QOL

Variable	Physical domain	Psychological domain	Social domain	Environment domain
	ß [95%CI]	ß [95%CI]	ß [95%CI]	ß [95%CI]
Intercept	*79.36 [75.20, 83.51]	*66.5 [62.92, 70.12]	*78.39 [73.21, 83.62]	*78.40 [74.01, 82.83]
**Age**				
< 40 years	Ref	Ref	Ref	Ref
≥ 40 years	-1.05 [-3.01, 0.82]	-0.08 [-1.74, 1.51]	-0.78 [-3.21, 1.62]	0.87 [-1.21, 2.92]
**Gender**				
Male	*4.23 [2.71, 5.82]	0.94 [-0.43, 2.32]	*2.78 [0.93, 4.74]	0.61 [-1.012, 2.23]
Female	Ref	Ref	Ref	Ref
**Residence**				
Rural	Ref	Ref	Ref	Ref
Urban	0.62 [-1.53, 2.80]	-0.53 [-2.44, 0.91]	-0.89 [-3.50, 1.81]	1.95 [ -0.31, 4.23]
**Country income level**				
Low	-0.77 [-3.40, 1.81]	-1.05 [-3.30, 1.21]	*5.85 [2.62, 9.13]	*-4.14 [-6.90, -1.32]
Low-middle	*-3.79 [-5.92, -1.73]	-1.06 [-2.82, 0.71]	-2.20 [-4.81, 0.43]	*-10.25 [-12.42, -8.14]
High-middle	*-2.95 [-4.95, -0.92]	-0.8 [-2.52, 0.93]	*-3.57 [-6.10, -2.12]	*-12.46 [-14.61, -10.30]
High	Ref	Ref	Ref	Ref
**Marital Status**				
Married	Ref	Ref	Ref	Ref
Single	0.57 [-1.00, 2.21]	-0.58 [-1.91, 0.72]	*-26.21 [ -28.21, -24.32]	-0.93 [-2.61, 0.73]
**Chronic disease**				
No	Ref	Ref	Ref	Ref
Yes	*-9.02 [-10.62, -7.44]	*-3.15 [-4.52, -1.82]	*-4.11 [-6.13, -1.11]	*-3.66 [-5.32, -1.91]
**Education**				
Less than primary	Ref	Ref	Ref	Ref
Primary/Secondary	*-2.38 [-4.41, -0.05]	-1.36 [-3.21, 0.53]	-0.54 [-3.21, 2.22]	*-3.43 [-5.71, -1.13]
University level	-0.43[-2.41, 1.51]	1.38 [-0.31, 3.12]	-0.15 [-2.61, 2.32]	0.57 [-1.53, 2.64]
Postgraduate Level	-0.68[-3.22, 1.80]	*2.57 [0.41, 4.82]	-1.16 [-4.30, 2.01]	0.48 [-2.21, 3.22]
**Working sector**				
Government sector	Ref	Ref	Ref	Ref
Not working	-2.38[-4.91, 0.22]	-2.10 [-4.33, 0.12]	-3.12 [-6.32, 0.03]	*-2.88 [-5.61, -0.22]
Private sector	-0.52[-2.3, 1.3]	-0.86 [-2.41, 0.72]	-1.96 [-4.21, 0.33]	-1.59 [-3.51, 0.32]
**Profession**				
Not working/housewife	-1.93[-4.34, 0.32]	0.32 [-1.62, 2.21]	-1.41 [-4.23, 1.44]	0.35 [-2.11, 2.81]
Non-Physician	Ref	Ref	Ref	Ref
**Years of work experience**				
≥ 15 years	*3.25[0.83, 5.73]	*3.19 [1.14, 5.33]	2.26 [-0.71, 5.32]	2.04 [-0.51, 4.52]
10–14 years	Ref	Ref	Ref	Ref
5–9 years	1.09 [-1.51, 3.72]	0.13 [-2.11, 2.34]	-0.38 [-3.61, 2.81]	-1.34 [-4.12, 1.43]
Less than 5 years	1.18 [-1.34, 3.64]	0.56 [-1.52, 2.61]	-1.98 [-5.01, 1.03]	-0.09 [-2.71, 2.44]
**Income-per-capita**				
Enough save	Ref	Ref	Ref	Ref
Enough	*-4.16[-5.91, -2.40]	*-3.52 [-4.91, -1.92]	*-3.60 [-5.80, -1.41]	*-9.11 [-11.03, -7.21]
Not enough +	*-9.52[-11.80, -7.31]	*-8.84 [-10.81, -6.90]	*-7.39 [-10.21, -4.64]	*-18.69 [-21.11, -16.32]
Not enough + +	*-17.44[-20.91, -14.04]	*-10.31 [-13.22, -7.44]	*-11.17 [-15.41, -6.92]	*-27.39 [-31.00, -23.84]
Not enough + + +	*-11.10[-14.22, -8.13]	*-9.25 [-11.91, -6.62]	*-7.75 [-11.6, -3.9]	*-24.63 [-27.91, -21.42]
**Pervious COVID-19 infections**				
No	Ref	Ref	Ref	Ref
Yes	*-2.98[-4.41, -1.60]	*-1.65 [-2.83, -0.41]	-1.73 [-3.5, 0.02]	*-1.67[-3.22, -0.21]
**Relative died due to COVID-19**				
No	Ref	Ref	Ref	Ref
Yes	*-1.56[-3.01, -0.12]	-0.03 [-1.21, 1.22]	-1.76 [-3.6, 0.04]	*-1.60 [-3.12, -0.06]
**COVID-19 vaccination**				
Fully vaccinated	Ref	Ref	Ref	Ref
Partially vaccinated	-0.32[-2.01, 1.32]	0.82 [-0.61, 2.34]	0.01 [-2.13, 2.13]	-0.77 [-2.16, 1.022]
Not Vaccinated	-0.90[-3.14, 1.23]	0.47 [ -1.41, 2.32]	-0.52 [-3.21, 2.14]	-0.52 [-2.83, 1.71]
Model statistics				
F -statistics (df)	19.87 (25,1982)	9.96 (25, 1982)	51.99 (25, 1982)	35.1 (25, 1982)
*p*-value	< 0.0001	< 0.0001	< 0.0001	< 0.0001
Adjusted R^2^	19.03%	10.04%	38.84%	29.81%

+ : Not enough and borrowing small amounts, +  + Not enough and borrowing large sums, +  +  + Not enough and in debt and can’t fulfill * :Signficant

## Discussion

The study findings indicate that a small percentage of participants achieved good scores in different domains of QoL. Specifically, the physical domain had the highest percentage of participants with good scores (42.7%), followed by the social domain (32.9%). On the other hand, the psychological and environmental domains had relatively lower percentages of participants with good scores (28.6% and 14.3% respectively). Moreover, the findings of this study suggest that certain individuals were more susceptible to experiencing poor QoL during the pandemic. This vulnerability was attributed to various factors, including socio-demographic background, financial status, and prior experience with COVID-19. These associations were confirmed through the implementation of regression analysis. These findings emphasize the enduring influence of the COVID-19 pandemic on the general population in Arab countries. Consequently, additional endeavors are necessary to relief the impact of COVID-19 and its consequences on QoL.

### Quality of life domains

In the context of the study, 57.3% had poor physical domain of QoL. While our study did not specifically assess participants' physical activity during the COVID-19 pandemic, it is worth noting that increasing physical activity can have positive effects on physical health and QoL. Engaging in regular exercise, even at home with family and friends, may offer several benefits during periods of boredom and disrupted daily routines [29]. We found that 71.4% of the Arab population had poor QoL in the psychological domain. We speculate that the high prevalence rates of anxiety, depression, stress, and insomnia reported by 48.9%, 50.6%, 41.4%, and 72.1% of respondents in Arab countries highlight the significant burden of mental health conditions in this population. It is well reported these conditions can have a negative impact on psychological domain of QoL [30]. In the current study, 67.1% had poor social QoL. We think that preventive measures like social distancing measures and restrictions on organizing and attending social activities as preventive measures to control the spread of COVID-19 had a negative impact on the social health. We found that 85.7% had poor environmental QoL. In fact, the pandemic highlighted and exacerbated existing socioeconomic disparities, with marginalized and vulnerable populations being disproportionately affected. Similarly, various studies have reported consistent negative effects of COVID-19 on the population. In Egypt, the pandemic significantly impacted the overall QoL of the public, particularly in terms of general well-being [24]. Dale et al. [31] discovered that in Australia, all domains of QoL have experienced a decline since the beginning of the pandemic, and this downward trend has persisted throughout the first year. Similarly, Persson et al. [32], reported a decrease in average QoL among the adult Swedish population from February to April 2020. The main contributing factor to this decline in QoL appears to be due to economic concerns and worries.

### Determinant of QoL among Arab population

#### Gender and marital status

In the current study, male gender was significantly associated with higher physical and social domains. Indeed, many studies in the region have reported lower QoL among females. For example, a study conducted by Mohsen and colleagues [24] in Egypt reported that the QoL of females was more affected compared to males. Studies carried out inTurkey [33] and Indonesia [34] reported conclusions that were comparable. On the other hand, in a study conducted in Germany, men tend to report higher levels of psychological and physical QoL compared to women. While women report higher levels of social QoL compared to men [35]. They suggest that females may face unique challenges and experiences that can negatively affect their overall well-being during the pandemic. We found that being married significantly affected the social domain of QoL. Previous studies have suggested that individuals who are married or in stable long-term partnerships tend to experience better health and higher life satisfaction. [36]. Likewise, Purba et al., [34] found that married Indonesian reported better QoL in almost all domains. There was a significant positive correlation between QoL and marriage length, indicating that longer marriages were associated with higher QoL scores. Living with one's spouse during the COVID-19 quarantine was found to be associated with lower distress levels. The presence of a spouse provided a sense of protection and support, contributing to better psychological well-being for the respondents [37].

#### Educational level and occupation

We found that working in the governmental sector had significantly affected the environmental domain while having working experience for more than 15 years significantly improved the physical and social domains. In fact, unemployment has consistently been shown to have a negative impact on well-being and life satisfaction, independent of the pandemic [38]. Similarly, the COVID-19 pandemic in Germany has impacted daily life significantly. Using data from the CORONA HEALTH App study, researchers analyzed the QoL of adults during the pandemic. Job seekers, those with reduced work hours and those who could not pursue their regular jobs experienced lower QoL [39]. In this study, level of education negatively affected all the domains of QoL except the psychological one. Similarly, except for the social domain, Indonesians with a higher level of education reported better QoL across nearly all areas [34]. Eicher et al. [35], found that individuals with higher levels of education reported a higher QoL in all domains compared to those with lower educational attainment. Moreover, these associations have been observed in studies conducted both during and outside of the pandemic [40]. Therefore, it is not surprising that educational status is linked to QoL during the pandemic as well.

#### Chronic disease

Individuals who reported chronic illnesses experienced a lower QoL across all domains compared to those without pre-existing condition. Indeed, the findings of Hammouda and colleagues [41] align with the present study, indicating that individuals with health conditions such as tuberculosis experience lower scores in all domains of QoL compared to the general population. This suggests that health conditions can significantly impact different aspects of individuals' well-being, including the physical, psychological, social, and environmental dimensions. On the other hand, Rubina et al. [42], reported that there was no significant differences between individuals with existing health conditions and those without such conditions, except for the domains of mobility and usual activities.

#### Income-per-capita

According to this study, a low income had a negative impact on all domains of QoL, whereas the country's income level negatively affected all domains except for the psychological domain. Likewise, Diener et al. [43], indicated a positive association between economic prosperity and QoL. The researchers found that wealth was significantly associated with 26 out of the 32 indicators examined in their study, suggesting that wealthier countries tend to have better QoL. On the other hand, Li et al. [44], reported that individuals living in communities with a higher average annual income were more likely to express greater concern for infectious diseases and pollution crises. These findings highlight the challenges faced by individuals in these circumstances regarding financial resources, access to healthcare and social services, living conditions, and overall environmental.

#### Previous COVID-19 infection

Significant variations were observed in specific domains of QoL among individuals who had previously contracted COVID-19, namely the physical, psychological, and environmental domains. Despite using different tool, Algamdi [45] reported similar finding among Saudi population who got COVID-19 infection. The management of COVID-19 indeed, involves a mandatory period of quarantine lasting 10–14 days, during which individuals have limited or no social interaction. This prolonged isolation contributes to a high prevalence of loneliness among those who experience symptoms related to COVID-19. Consequently, the deterioration in QoL can be attributed to the adverse effects of loneliness stemming from the quarantine measures.

#### COVID-19 vaccination

In an intriguing finding, there was a significant association between COVID-19 vaccination and the social and environmental domains of QoL in bivariable analysis. Nevertheless, when considering multivariate regression analysis, this association was no longer statistically significant. Extensive research and clinical trials have demonstrated that COVID-19 vaccines are proven to be safe, with robust immunogenicity [46], efficacy, and effectiveness [47]. However, high rates of vaccine hesitancy in the region towards the primary series of vaccination [48] or booster doses [49] may explain the insufficient trust in vaccine protection that is reflected on population QoL [50]. On the other hand, Montero-López et al. [51] found that among patients with systematic immune diseases, the unvaccinated group exhibited poorer scores in various aspects of their psychological well-being and perceived lower physical health compared to the vaccinated group. Specifically, the unvaccinated group experienced a decline in the overall QoL, facing challenges in mobility and performing household tasks, experiencing more pain or discomfort, and reporting higher levels of anxiety and depression.

#### Death of relative due to COVID-19

In the bivariate analysis, the occurrence of death among relatives from a COVID-19 infection had a significant impact on all domains of QoL, except for the psychological domain. However, in the multivariate analysis, after considering other factors, the effect of death among relatives remained significant only in the physical and environmental domains of QoL. The effect of COVID-19 pandemic on relatives was reported by the study conducted by Rubina eta al. [42]. As per our finding, family members reported that their relative's COVID-19 had an impact on their sexual life, particularly among males. A similar finding was reported in Brazil, indicating that the loss of a family member or friend directly due to COVID-19 intensifies psychological distress. This effect appears to be significantly heightened among individuals with a pre-existing history of mental disorders [52]. Indeed, the highlighted finding underscores the importance of implementing interventional programs specifically targeting relatives of individuals who have passed away due to COVID-19. These programs can play a crucial role in providing early prevention strategies to address psychological distress and ultimately improve the QoL for this population.

### Limitations and strengths

There are a few limitations to consider in our study. Firstly, there is a potential for selection bias as some individuals may have limited access to specific social media platforms or online communities that were used to distribute our survey. However, it is worth noting that internet usage in the Middle East and North Africa region is quite high, with nearly 80.0% of the population reported to be using the internet, and the use of social media has significantly increased in recent years within EMR countries. Secondly, the reliance on self-reported data introduces the possibility of recall and social desirability biases. Participants may not accurately remember certain details or may provide responses that are more socially acceptable rather than entirely truthful. Furthermore, our sample selection method was non-probability based, which means that our finding may not be generalizable to the entire population. Lastly, it is important to acknowledge that cross-sectional surveys inherently come with limitations, including the inability to establish causality between variables. However, this study has several strengths that contribute to its overall value and reliability. Firstly, it adopts a cross-sectional design, allowing for the collection of data from a large sample of Arab adults from different countries. This broad representation enhances the study's ability to capture a diverse range of perspectives and experiences related to the COVID-19 pandemic's impact on QoL. Secondly, the study utilizes an anonymous online survey, which can encourage participants to provide honest and uninhibited responses. Lastly, the study employs the short version of the World Health Organization QoL instrument, a well-established and validated tool for assessing QoL in three languages ensured the reliability and comparability of its findings with existing research in this field.

## Conclusions

Our finding provides valuable insights on the importance of improving the QoL for the general population in the Arab world and the necessity to promote community resilience and recovery after the pandemic. It highlights that it is crucial to prioritize socially disadvantaged groups who have experienced significant declines in their QoL, and to develop response strategies that cater to their specific needs. The lessons learned from the ongoing pandemic underscore the significance of establishing preventive measures and community preparedness to ensure both physical and mental readiness for future outbreaks of infectious diseases. Strategies and interventions such as strengthening the healthcare system and improving access through proper investments and reorientation of resources; improving mental health services and support; health education and promotion actions; fostering social support actions and networks; promoting economic recovery and building local governance, partnership and collaboration are all needed to improve QoL of populations post COVID-19 and for enabling readiness for any future pandemics or disasters.

## Supplementary Information

**Additional file 1**.

## Data Availability

The datasets used and analyzed during the current study are available from the corresponding author on reasonable request.

## Abbreviations

CI: Confidence interval
COVID-19**:**: Coronavirus disease 2019
EMR: Eastern Mediterranean Region
HRQoL: Health-Related Quality of Life
PHEIC: Public Health Emergency of International Concern
QoL: Quality of life
SD: Standard deviation
SPSS: Statistical Packages for Social Sciences
WHO: World Health Organization
