# Supplementary material for: Quality of life among the Arab population two years after COVID-19 pandemic
Source: BMC Public Health. 2023 Jun 30;23:1268. doi: 10.1186/s12889-023-16171-z (PMC10311886; doi:10.1186/s12889-023-16171-z)
Supplement: Supplementary file 1 — Additional file 1. [file 12889_2023_16171_MOESM1_ESM.docx]

Assessment of quality of life among individuals residing in the Arab world.

This questionnaire is about your health and aspects of your life. Please answer all* questions. If you are not sure which answers you want to choose, try to choose the answer that best suits you and that can be considered your 1^st^ answer. You must take into account all your standards, hopes, pleasures, and interests. We ask you to think about your life over the past two weeks. The questionnaire does not require entering any data indicating your .identity, your data will only be used for research purposes.

1. **Select your preferred language:**

- English go to Page 1
- اللغة العربية go to Page 7
- Francaise go to page12

**Personal data**

1. **Sex**
   1. Male
   2. Female
2. **Nationality**
   1. Egypt
   2. Saudi Arabia
   3. United Emirates
   4. Kuwait
   5. Bahrain
   6. Oman
   7. Qatar
   8. Jordan
   9. Iraq
   10. Syria
   11. Lebanon
   12. Yemen
   13. Palestine
   14. Libya
   15. Algeria
   16. Tunisia
   17. Morocco
   18. Mauritania
   19. Somalia
   20. Sudan
   21. Other:
3. **Country of residency?**
4. Egypt
5. Saudi Arabia
6. United Emirates
7. Kuwait
8. Bahrain
9. Oman
10. Qatar
11. Jordan
12. Iraq
13. Syria
14. Lebanon
15. Yemen
16. Palestine
17. Libya
18. Algeria
19. Tunisia
20. Morocco
21. Mauritania
22. Somalia
23. Sudan
24. **Age**
25. 18-30 years
26. 31-40 years
27. 41-50 years
28. 51-60 years
29. 61-70 years
30. > 70 years
31. **Marital status**
32. Married
33. Single
34. Widow
35. Divorced
36. **Education level**
37. Illiterate
38. Reads and writes
39. Primary
40. Preparatory
41. Secondary
42. University graduate
43. Postgraduate
44. **Work sector**
    1. Governmental
    2. Private
    3. Not-working
45. **profession**
46. Non-Healthcare worker
47. Not working or housewife
48. **Your experience**
49. Less than 5 years
50. 5- ‹10 years
51. 10 - ‹15 years
52. ≥ 15 years
53. **Do you suffer from a chronic disease or disability?**
54. Yes
55. No

**Assessment of the economic and social situation of the family**

1. **Per capita household income**
2. Not enough and he is in debt and he cannot fulfill the debt
3. Not enough and borrowing large sums
4. Not enough and borrowing small amounts
5. It is enough
6. Enough and save
7. **Number of family members**
8. ≥7
9. 6
10. 5
11. <5
12. **Number of rooms in the house (without the hall, kitchen, and bathroom)**
13. 1
14. 2
15. 3
16. 4
17. 5
18. >5

**COVID infection data**

1. **Have you been infected with Coronavirus?**
2. Yes
3. No
4. **What Situation of receiving the COVID-19 vaccine?**
5. Not vaccinated
6. Partially vaccinated.
7. Fully vaccinated.
8. Received the booster dose.
9. **Has any of your relatives died of coronavirus?**
10. Yes
11. No
12. **If you did not receive the vaccination yet, did this lead to preventing you from entering government institutions or public places?**
13. Yes
14. No

**General assessment**

These questions are completely away from the religious and faith-based meaning of the concept of satisfaction, but rather target your general assessment of your life/if you are convinced of it or would like to change some things to become better than they are now.

1. **How would you rate your quality of life?**
   1. Very poor
   2. Poor
   3. Neither poor nor good
   4. Good
   5. Very good
2. **How satisfied are you with your health?**
   1. Very dissatisfied
   2. Dissatisfied
   3. either satisfied nor dissatisfied
   4. Satisfied
   5. Very satisfied

**The following questions ask about how much you have experienced certain things in the last two weeks.**

1. **To what extent do you feel that physical pain prevents you from doing what you need to do?**
   1. Not at all
   2. A little
   3. A moderate amount
   4. Very much
   5. An extreme
2. **How much do you need any medical treatment to function in your daily life?**

   2. Not at all
   3. A little
   4. A moderate amount
   5. Very much
   6. An extreme
3. **How much do you enjoy your life?**

   2. Not at all
   3. A little
   4. A moderate amount
   5. Very much
   6. An extreme
4. **How much do you enjoy your life?**
   1. Not at all
   2. A little
   3. A moderate amount
   4. Very much
   5. An extreme
5. **How well are you able to concentrate?**
   1. Not at all
   2. A little
   3. A moderate amount
   4. Very much
   5. An extreme
6. **How safe do you feel in your daily life?**
   1. Not at all
   2. A little
   3. A moderate amount
   4. Very much
   5. An extreme
7. **How healthy is your physical environment?**
   1. Not at all
   2. A little
   3. A moderate amount
   4. Very much
   5. An extreme
8. **Do you have enough energy for everyday life?**
   1. Not at all
   2. A little
   3. Moderately
   4. Mostly
   5. Completely
9. **Are you able to accept your bodily appearance?**
   1. Not at all
   2. A little
   3. Moderately
   4. Mostly
   5. Completely
10. **Do you have enough money to meet your needs?**
    1. Not at all
    2. A little
    3. Moderately
    4. Mostly
    5. Completely
11. **How available to you is the information that you need in your day-to-day life?**
    1. Not at all
    2. A little
    3. Moderately
    4. Mostly
    5. Completely
12. **To what extent do you have the opportunity for leisure activities?**
    1. Not at all
    2. A little
    3. Moderately
    4. Mostly
    5. Completely
13. **How well are you able to get around?**
    1. Very poor
    2. Poor
    3. Neither poor nor good
    4. Good
    5. Very good

**The following questions ask you to say how good or satisfied you have felt about various aspects of your life over the last two weeks.**

1. **How satisfied are you with your sleep?**
   1. Very dissatisfied
   2. Dissatisfied
   3. Neither satisfied nor dissatisfied
   4. Satisfied
   5. Very satisfied
2. **How satisfied are you with your ability to perform your daily living activities?**
   1. Very dissatisfied
   2. Dissatisfied
   3. Neither satisfied nor dissatisfied
   4. Satisfied
   5. Very satisfied
3. **How satisfied are you with your capacity for work?**
   1. Very dissatisfied
   2. Dissatisfied
   3. Neither satisfied nor dissatisfied
   4. Satisfied
   5. Very satisfied
4. **How satisfied are you with yourself?**
   1. Very dissatisfied
   2. Dissatisfied
   3. Neither satisfied nor dissatisfied
   4. Satisfied
   5. Very satisfied
5. **How satisfied are you with your personal relationships?**
   1. Very dissatisfied
   2. Dissatisfied
   3. Neither satisfied nor dissatisfied
   4. Satisfied
   5. Very satisfied
6. **How satisfied are you with your sex life?**
   1. Very dissatisfied
   2. Dissatisfied
   3. Neither satisfied nor dissatisfied
   4. Satisfied
   5. Very satisfied
7. **How satisfied are you with the support you get from your friends?**
   1. Very dissatisfied
   2. Dissatisfied
   3. Neither satisfied nor dissatisfied
   4. Satisfied
   5. Very satisfied
8. **How satisfied are you with the conditions of your living place?**
   1. Very dissatisfied
   2. Dissatisfied
   3. Neither satisfied nor dissatisfied
   4. Satisfied
   5. Very satisfied
9. **How satisfied are you with your access to health services?**
   1. Very dissatisfied
   2. Dissatisfied
   3. Neither satisfied nor dissatisfied
   4. Satisfied
   5. Very satisfied
10. **How satisfied are you with your transport?**
    1. Very dissatisfied
    2. Dissatisfied
    3. Neither satisfied nor dissatisfied
    4. Satisfied
    5. Very satisfied

**The following question refers to how often you have felt or experienced certain things in the last**

**two weeks.**

1. **How often do you have negative feelings such as blue mood, despair, anxiety, depression?**
   1. Never
   2. Seldom
   3. Quite often
   4. Very often
   5. Always

**تقييم جودة الحياة لدى الأفراد المقيمين داخل الوطن العربي**

هذا الإستبيان يتعلق بمدى صحتك  والجوانب المحيطة  بحياتك. من فضلك أجب على كل الأسئلة . إذا لم تكن متأكدا من الإجابات التى تريد إختيارها ‘ حاول أن تختار الإجابة التى تلائمك أكثر والتى يمكن إعتبارها إجابتك الأولى. يجب أن تضع فى الحسبان كل ما لديك من معايير ‘  وآمال ومَسرات وإهتمامات . إننا نسألك أن تفكر فى حياتك خلال الأسبوعين الماضيين.هذا الاستبيان لن يطلب اى معلومات تدل على هويتك ولن يتم استخدام بياناتك سوى لغرض البحث.

**البيانات الشخصية:**

1. **النوع /الجنس**:
   1. ذكر
   2. انثى
2. **الجنسية**
   1. مصر
   2. السعودية
   3. الامارات
   4. الكويت
   5. البحرين
   6. عمان
   7. قطر
   8. الاردن
   9. العراق
   10. سوريا
   11. لبنان
   12. اليمن
   13. فلسطين
   14. ليبيا
   15. الجزائر
   16. تونس
   17. المغرب
   18. موريتنيا
   19. الصومال
   20. السودان
   21. أخرى:
3. **بلد  الإقامة**
   1. مصر
   2. السعودية
   3. الامارات
   4. الكويت
   5. البحرين
   6. عمان
   7. قطر
   8. الاردن
   9. العراق
   10. سوريا
   11. لبنان
   12. اليمن
   13. فلسطين
   14. ليبيا
   15. الجزائر
   16. تونس
   17. المغرب
   18. موريتنيا
   19. الصومال
   20. السودان
4. **العمر**
   1. 18-30 عام
   2. 31-40 عام
   3. 41-50 عام
   4. 51-60عام
   5. 61-70عام
   6. اكبر من 70 عام
5. **الحالة الاجتماعية**
   1. متزوج/ة
   2. اعزب /انسة
   3. ارمل/ة
   4. مطلق/ة
6. **مستوى التعليم:**
   1. أمي لا يقرأ ولا يكتب
   2. يقرأ ويكتب
   3. ابتدائي
   4. اعدادى
   5. ثانوى
   6. جامعى
   7. دراسات عليا
7. **محل العمل**:
   1. قطاع عام او حكومة
   2. قطاع خاص
   3. لا اعمل
8. **التخصص**
   1. اعمل خارج المجال الصحي
   2. لا اعمل او ربة منزل
9. **سنوات الخبرة**
   1. اقل من 5سنوات
   2. 5 - ‹ 10 سنوات
   3. 10 - ‹ 15سنة
   4. ≥ 15 سنة
10. **هل تعاني من احد الامراض المزمنة او الاعاقات الجسدية؟**
    1. نعم
    2. لا

**تقييم الحالة الاقتصادية والاجتماعية للأسرة**

1. **نصيب الفرد من دخل الاسرة**
   1. لا يكفى ويستدين ولا يستطيع الوفاء بالدين
   2. لا يكفى ويستدين بمبالغ كبيرة
   3. لا يكفى ويستدين بمبالغ صغيرة
   4. يكفى
   5. يكفى ويدخر
2. **عدد افراد الاسرة**
   1. ≥7
   2. 6
   3. 5
   4. 5˂
3. **عدد غرف المنزل (بدون الصالة و المطبخ والحمام)**
   1. 1
   2. 2
   3. 3
   4. 4
   5. 5
   6. >5

**تاريخ الاصابة بفيرس كوفيد**

1. **هل تأكد اصابتك بمرض كوفيد 19 من قبل؟**
   1. نعم
   2. لا
2. ا**لموقف من التطعيم / اللقاح المضاد لمرض كوفيد 19**
   1. لم اتلقى اللقاح
   2. تطعيم جزئي
   3. اتممت جرعات اللقاح
   4. تلقيت الجرعة المنشطة
3. **هل توفي احد اقاربك متاثرا بإصابته بمرض كوفيد 19**
   1. نعم
   2. لا
4. **ان كنت لم تتلقى التطعيم بعد، هل أدى ذلك لمنعك من دخول مؤسسات حكومية او اماكن عامة ؟**
   1. نعم
   2. لا

**التقييم العام**

هذه الاسئلة بعيدة تماما عن المعنى الدينى والايمانى لمفهوم الرضا بل تستهدف تقييمك العام لحياتك/ ان كنت مقتنعا بها او تود تغيير بعض الاشياء لتصبح افضل مما هي عليه الان

1. **كيف تقيم جودة حياتك؟**
   1. ضعيفة جدا
   2. ضعيفة
   3. متوسط
   4. جيدة
   5. جيدة جدا
2. **إلى أي مدى انت مقتنع (راضٍ) عن صحتك؟**
   1. غير راضٍ تماما
   2. غير راضٍ
   3. راضٍ نوعا ما
   4. مقتنع
   5. مقتنع تماما

**الأسئلة التالية تستفسر عن مدى حجم أشياء معينة تعرضت لها خلال الإسبوعين الماضيين**

:

1. **إلى أى مدى تشعر أن الألم الجسمانى يمنعك من القيام بأداء شىء تحب تأديته؟**
   1. لم اشعر بذلك ابدا
   2. قليلاً
   3. إلى حد متوسط
   4. كثيرا جداً
   5. إلى أبعد الحدود
2. **إلى أى مدى تحتاج إلى العلاج الطبى حتى تؤدى وظيفتك اليومية؟**
   1. لم احتاجها ابدا
   2. قليلاً
   3. إلى حد متوسط
   4. كثيرا جداً
   5. إلى أبعد الحدود
3. **إلى أى مدى تستمتع بالحياة؟**
   1. مطلقا
   2. قليلاً
   3. إلى حد متوسط
   4. كثيرا جداً
   5. إلى أبعد الحدود
4. **إلى أى مدى تشعر أن حياتك ذات قٌيمة؟**
   1. لم اشعر بذلك ابدا
   2. قليلاً
   3. إلى حد متوسط
   4. كثيرا جداً
   5. إلى أبعد الحدود

1. **إلى أى مدى أنت قادر على التركيز؟**
   1. لا استطيع التركيز مطلقا
   2. قليلاً
   3. إلى حد متوسط
   4. كثيرا جداً
   5. إلى أبعد الحدود
2. **إلى أى مدى تشعر بالأمان فى حياتك اليومية؟**
   1. لا اشعر بالامان نهائيا
   2. قليلاً
   3. إلى حد متوسط
   4. كثيرا جداً
   5. إلى أبعد الحدود
3. **إلى أى مدى تشعر بملاءمة البيئة المحيطة بك؟**
   1. غير ملائمة تماما
   2. قليلاً
   3. إلى حد متوسط
   4. كثيرا جداً
   5. إلى أبعد الحدود
4. **هل لديك الطاقة الكافية لممارسة حياتك اليومية؟**
5. لايوجد
6. قليلاً
7. متوسط
8. فوق المتوسط
9. تماما
10. **هل أنت قادر على قبول مظهرك الجسمانى؟**
    1. لا أقبله
    2. قليلاً
    3. متوسط
    4. فوق المتوسط
    5. تماما
11. **هل لديك المال الكافى لتلبية إحتياجاتك؟**
    1. لايوجد
    2. قليلاً
    3. متوسط
    4. فوق المتوسط
    5. تماما
12. **إلى أى مدى تتاح لديك المعلومات التى تحتاجها فى حياتك اليومية ؟**
    1. لايوجد
    2. قليلاً
    3. متوسط
    4. فوق المتوسط
    5. تمام
13. **إلى أى مدى تتاح لديك الفرصة للأنشطة الترويحية عند الفراغ؟**
    1. لايوجد
    2. قليلاً
    3. متوسط
    4. فوق المتوسط
    5. تماما
14. **ما هى مقدرتك الصحية على التحرك ؟**
    1. قليلا جدا
    2. قليلا
    3. متوسط
    4. جيد
    5. جيد جدا

**الأسئلة التالية تستفسر عن مدى شعورك بالإستحسان أو الرضا عن جوانب متعددة فى حياتك خلال الإسبوعين الماضيين:**

1. **إلى أى مدى تشعر بالإرتياح فى نومك؟**
2. غير راض تماما
3. غير راض
4. متوسط
5. راض
6. راض جدا
7. **إلى أى مدى أنت راض عن مقدرتك فى أداء أنشتطك اليومية؟**
8. غير راض تماما
9. غير راض
10. متوسط
11. راض
12. راض جدا
13. **إلى أى درجة أنت راض عن مقدرتك فى أداء عملك؟**
14. غير راض تماما
15. غير راض
16. متوسط
17. راض
18. راض جدا
19. **إلى أى مدى أنت راض عن نفسك؟**
20. غير راض تماما
21. غير راض
22. متوسط
23. راض
24. راض جدا
25. **إلى أى مدى أنت راض عن علاقاتك الشخصية؟**
26. غير راض تماما
27. غير راض
28. متوسط
29. راض
30. راض جدا
31. **إلى أى مدى أنت راض عن حياتك الجنسية؟**
32. غير راض تماما
33. غير راض
34. متوسط
35. راض
36. راض جدا
37. غير متزوج
38. إ**لى أى مدى أنت راض عن المساندة التى تجدها من أصدقائك؟**
39. غير راض تماما
40. غير راض
41. متوسط
42. راض
43. راض جدا
44. **إلى أى مدى أنت راض عن حالة المكان الذى تعيش فيه؟**
45. غير راض تماما
46. غير راض
47. متوسط
48. راض
49. راض جدا
50. **إلى أى مدى أنت راض عن حصولك على الخدمات الصحية؟**
51. غير راض تماما
52. غير راض
53. متوسط
54. راض
55. راض جدا
56. **إلى أى مدى أنت راض عن توفر وسائل النقل لديك؟**
57. غير راض تماما
58. غير راض
59. متوسط
60. راض
61. راض جدا

**السؤال التالى يستفسر عن مدى شعورك أو تجربتك للقيام بأشياء معينة خلال الإسبوعين الماضيين:**

1. **إلى أى مدى تنتابك مشاعر سلبية مثل الحزن واليأس والقلق والإكتئاب؟**
   1. أبدا
   2. نادرا
   3. أحيانا
   4. في معظم الأحيان
   5. دائما

**Évaluation de la qualité de vie des individus. résidant dans le monde arabe**

Ce sondage porte sur votre santé et les aspects de votre vie. Veuillez répondre à* toutes les questions. Si vous n'êtes pas sûr des réponses que vous voulez choisir, essayez de choisir la réponse qui vous convient le mieux et qui peut être considérée comme votre première réponse. Vous devez tenir compte de toutes vos normes, espoirs, plaisirs et intérêts. Nous vous demandons de réfléchir à votre vie au cours des deux dernières semaines.Ce sondage ne demandera aucune information indiquant votre.identité et vos informations personnelles ne seront utilisées qu'à des fins de recherché.

**Données Personnelles**

1. **Sexe**
   1. homme
   2. femme
2. **Nationalité**
   1. Egyptiennne
   2. Saoudienne
   3. Emiratie
   4. Koweitienne
   5. Bahreinienne
   6. Omanaise
   7. Qatarienne
   8. Jordanienne
   9. Iraquienne
   10. Syrienne
   11. Libanaise
   12. Yémenite
   13. Palestinienne
   14. Libyenne
   15. Algérienne
   16. Tunisienne
   17. Marocaine
   18. Mauritanienne
   19. Somalienne
   20. Soudanaise
   21. autre:
3. **Pays de résidence**
   1. Egypte
   2. Arabie Saoudite
   3. Emirats Unis
   4. Kweit
   5. Bahrain
   6. Oman
   7. Qatar
   8. Jordan
   9. Iraq
   10. Syria
   11. Liban
   12. Yémen
   13. Palestine
   14. Libye
   15. Algérie
   16. Tunisie
   17. Maroc
   18. Mauritanie
   19. Somalie
   20. Soudan
4. **Âge**
   1. 18-30
   2. 31-40
   3. 41-50
   4. 51-60
   5. 61-70
   6. >70
5. **État civil**
   1. Marié/Mariée
   2. Célibataire
   3. Veuf/veuve
   4. Divorcée/Divorcé
6. **Niveau d'instruction**
   1. Analphabète
   2. Lit et écrit
   3. Primaire
   4. Préparatoire
   5. Secondaire
   6. License
   7. Diplôme d'études supérieures
7. **Secteur de travail**
   1. Secteur public
   2. Secteur privé
   3. Ca ne fonctionne pas
8. **Profession**
   1. Je travaille dans un domaine autre que la santé
   2. Je ne travaille pas ou femme au foyer
9. **Ton expérience**
   1. Moins de 5 ans
   2. 5- ‹10 ans
   3. 10 - ‹15 ans
   4. ≥ 15 ans
10. **Vous souffrez d’une maladie chronique ou d’un handicap ?**
    1. Oui
    2. Non

**Évaluation de la situation économique et sociale de la famille**

1. **Revenu des ménages par habitant**
   1. non suffisant
   2. non suffisant et emprunte de larges sommes d'argent
   3. non suffisant et emprunte de petites sommes d'argent
   4. suffisant
   5. suffisant et economise de l'argent
2. **Nombre de membres de la famille?**
   1. ≥ 7
   2. 6
   3. 5
   4. < 5
3. **Nombre de pièces de la maison (sans le hall, la cuisine et la salle de bain)**
   1. 1
   2. 2
   3. 3
   4. 4
   5. 5
   6. >5

**Données sur les infections Covid**

1. **Avez-vous déjà confirmé que vous avez le COVID -19 ?**
   1. Oui
   2. Non
2. **Situation de vaccination pour  COVID-19 ?**
   1. Non vacciné
   2. Partiellement vacciné
   3. Complétement vacciné
   4. A reçu la dose de rappel
3. **L'un de vos proches est-il décédé du coronavirus ?**
   1. Oui
   2. Non
4. **Si vous n'avez pas encore été vacciné, cela vous a-t-il empêché d'entrer dans les institutions gouvernementales ou lieux publics?**
   1. Oui
   2. Non

**Évaluation générale**

Ces questions sont complètement éloignées de la signification religieuse et confessionnelle du concept de satisfaction, mais ciblent plutôt votre appréciation générale de votre vie/si vous en êtes convaincu ou aimeriez changer certaines choses pour devenir meilleures qu'elles ne le sont actuellemen

1. **Comment trouvez-vous votre qualité de vie ?**
2. Très mauvaise
3. Mauvaise
4. Ni bonne, ni mauvaise
5. Bonne
6. Très bonne
7. **Dans quelle mesure êtes-vous satisfait de votre santé ?**
   1. Pas du tout satisfait
   2. Pas satisfait
   3. Ni satisfait ni insatisfait
   4. Satisfait
   5. Très Satisfait

**Les questions suivantes ont pour but de déterminer dans quelle mesure vous avez ressenti certaines choses au cours des deux dernières semaines**

1. **Dans quelle mesure la douleur (physique)vous empêche-t-elle de faire ce que vous avez à faire ?**
   1. Pas du tout
   2. Un peu
   3. Modérément
   4. Beaucoup
   5. Complètement
2. **Dans quelle mesure un traitement médical vous est-il nécessaire pour faire face à lavie de tous les jours ?**
   1. Pas du tout
   2. Un peu
   3. Modérément
   4. Beaucoup
   5. Complètement
3. **Dans quelle mesure trouvez-vous la vie agréable ?**
   1. Pas du tout
   2. Un peu
   3. Modérément
   4. Beaucoup
   5. Complètement
4. **Dans quelle mesure votre vie a-t-elle un sens ?**
   1. Pas du tout
   2. Un peu
   3. Modérément
   4. Beaucoup
   5. Complètement
5. **Dans quelle mesure êtes-vous capable de vous concentrer ?**
   1. Pas du tout
   2. Un peu
   3. Modérément
   4. Beaucoup
   5. Tout à fait
6. **Dans quelle mesure vous sentez-vous en sécurité dans votre vie de tous les jours ?**
   1. Pas du tout
   2. Un peu
   3. Modérément
   4. Beaucoup
   5. Tout à fait
7. **Dans quelle mesure votre environnement est-il sain (pollution, bruit, salubrité, etc.) ?**
   1. Pas du tout
   2. Un peu
   3. Modérément
   4. Beaucoup
   5. Tout à fait
8. **Avez-vous assez d'énergie dans la vie de tous les jours ?**
   1. Pas du tout
   2. Un peu
   3. Modérément
   4. Suffisamment
   5. Tout à fait
9. **Acceptez-vous votre apparence physique ?**
   1. Pas du tout
   2. Un peu
   3. Modérément
   4. Suffisamment
   5. Tout à fait
10. **Avez-vous assez d'argent pour satisfaire vos besoins ?**
    1. Pas du tout
    2. Un peu
    3. Modérément
    4. Suffisamment
    5. Tout à fait
11. **Avez-vous le sentiment d'être assez informé pour faire face à la vie de tous les jours ?**
    1. Pas du tout
    2. Un peu
    3. Modérément
    4. Suffisamment
    5. Tout à fait
12. **Dans quelle mesure avez-vous la possibilité d'avoir des activités de loisirs ?**
    1. Pas du tout
    2. Un peu
    3. Modérément
    4. Suffisamment
    5. Tout à fait
13. **Comment trouvez-vous votre capacité à vous déplacer seul ?**
    1. Pas du tout
    2. Un peu
    3. Modérément
    4. Bonne
    5. Tout à fait

**Les questions suivantes vous demandent dans quelle mesure vous avez été content(e) ou satisfait(e) de différents aspects de votre vie ces deux dernières semaines.**

1. **Dans quelle mesure êtes-vous satisfait de votre sommeil ?**
   1. Très insatisfait
   2. Insatisfait
   3. Ni satisfait ni insatisfait
   4. Satisfait
   5. Très satisfait
2. **Dans quelle mesure êtes-vous satisfait de votre capacité à accomplir vos activités quotidiennes ?**
   1. Très insatisfait
   2. Insatisfait
   3. Ni satisfait ni insatisfait
   4. Satisfait
   5. Très satisfait
3. **Dans quelle mesure êtes-vous satisfait de votre capacité à travailler ?**
   1. Très insatisfait
   2. Insatisfait
   3. Ni satisfait ni insatisfait
   4. Satisfait
   5. Très satisfait
4. **Dans quelle mesure avez-vous une bonne opinion de vous-même ?**
   1. Très insatisfait
   2. Insatisfait
   3. Ni satisfait ni insatisfait
   4. Satisfait
   5. Très satisfait
5. **Dans quelle mesure êtes-vous satisfait de vos relations personnelles**
   1. Très insatisfait
   2. Insatisfait
   3. Ni satisfait ni insatisfait
   4. Satisfait
   5. Très satisfait
6. **Dans quelle mesure êtes-vous satisfait de votre vie sexuelle ?**
   1. Très insatisfait
   2. Insatisfait
   3. Ni satisfait ni insatisfait
   4. Satisfait
   5. Très satisfait
   6. Célibataire
7. **Dans quelle mesure êtes-vous satisfait dusoutien que vous recevez de vos amis ?**
   1. Très insatisfait
   2. Insatisfait
   3. Ni satisfait ni insatisfait
   4. Satisfait
   5. Très satisfait
8. **Dans quelle mesure êtes-vous satisfait del'endroit où vous vivez ?**
   1. Très insatisfait
   2. Insatisfait
   3. Ni satisfait ni insatisfait
   4. Satisfait
   5. Très satisfait
9. **Dans quelle mesure avez-vous facilementaccès aux soins dont vous avez besoin ?**
   1. Très insatisfait
   2. Insatisfait
   3. Ni satisfait ni insatisfait
   4. Satisfait
   5. Très satisfait
10. **Dans quelle mesure êtes-vous satisfait devos moyens de transport ?**
    1. Très insatisfait
    2. Insatisfait
    3. Ni satisfait ni insatisfait
    4. Satisfait
    5. Très satisfait

**La question suivante concerne la fréquence à laquelle vous avez ressenti ou vécu certaines choses au cours des deux dernières semaines.**

1. **Éprouvez-vous souvent des sentiments négatifs comme le cafard, le désespoir, l'anxiété ou la dépression ?**
2. Jamais
3. Parfois
4. Souvent
5. Très souvent
6. Toujours
